# Daidzein Inhibits Muscle Atrophy by Suppressing Inflammatory Cytokine- and Muscle Atrophy-Related Gene Expression

**DOI:** 10.3390/nu16183084

**Published:** 2024-09-13

**Authors:** Chihiro Munekawa, Takuro Okamura, Saori Majima, Budau River, Sayaka Kawai, Ayaka Kobayashi, Hanako Nakajima, Nobuko Kitagawa, Hiroshi Okada, Takafumi Senmaru, Emi Ushigome, Naoko Nakanishi, Masahide Hamaguchi, Michiaki Fukui

**Affiliations:** Department of Endocrinology and Metabolism, Graduate School of Medical Science, Kyoto Prefectural University of Medicine, Kyoto 602-8566, Japan; c-mori@koto.kpu-m.ac.jp (C.M.);

**Keywords:** mice, high-fat diet, high-sucrose diet, sarcopenia, sarcopenic obesity, C2C12 myotubes, muscle atrophy, soy isoflavones, daidzein

## Abstract

Background: Sarcopenic obesity, which is associated with a poorer prognosis than that of sarcopenia alone, may be positively affected by soy isoflavones, known inhibitors of muscle atrophy. Herein, we hypothesize that these compounds may prevent sarcopenic obesity by upregulating the gut metabolites with anti-inflammatory effects. Methods: To explore the effects of soy isoflavones on sarcopenic obesity and its mechanisms, we employed both in vivo and in vitro experiments. Mice were fed a high-fat, high-sucrose diet with or without soy isoflavone supplementation. Additionally, the mouse C2C12 myotube cells were treated with palmitic acid and daidzein in vitro. Results: The isoflavone considerably reduced muscle atrophy and the expression of the muscle atrophy genes in the treated group compared to the control group (*Fbxo32*, *p* = 0.0012; *Trim63*, *p* < 0.0001; *Foxo1*, *p* < 0.0001; *Tnfa*, *p* = 0.1343). Elevated levels of daidzein were found in the muscles and feces of the experimental group compared to the control group (feces, *p =* 0.0122; muscle, *p =* 0.0020). The real-time PCR results demonstrated that the daidzein decreased the expression of the palmitate-induced inflammation and muscle atrophy genes in the C2C12 myotube cells (*Tnfa*, *p =* 0.0201; *Il6*, *p =* 0.0008; *Fbxo32*, *p <* 0.0001; *Hdac4*, *p =* 0.0002; *Trim63*, *p =* 0.0114; *Foxo1*, *p <* 0.0001). Additionally, it reduced the palmitate-induced protein expression related to the muscle atrophy in the C2C12 myotube cells (*Foxo1*, *p =* 0.0078; MuRF1, *p =* 0.0119). Conclusions: The daidzein suppressed inflammatory cytokine- and muscle atrophy-related gene expression in the C2C12 myotubes, thereby inhibiting muscle atrophy.

## 1. Introduction

Sarcopenia, characterized by an age-associated syndrome marked by the deterioration of muscle mass, muscle strength, and/or physical function [1], is closely associated with increased instability, falls, and frailty in elderly individuals. It is one of the leading causes of long-term care dependency [2]. Age-related muscle weakness, a hallmark of sarcopenia, is becoming increasingly prevalent globally [3,4]. This syndrome is associated with several adverse health outcomes, including increased all-cause mortality, cardiovascular disease, and mobility limitations [5,6]. Sarcopenic obesity, which involves both reduced muscle mass and increased visceral fat [7], presents a worse prognosis than that of sarcopenia alone [8,9,10]. The incidence of sarcopenic obesity has surged worldwide, driven largely by sedentary lifestyles and diets rich in fats and sugars, typical of processed foods [11]. Due to the lack of standardized diagnostic criteria, estimates of its prevalence vary widely. For instance, a Korean prospective cohort study of healthy volunteers aged 20 to 80 years reported incidence rates of sarcopenic obesity ranging from 0.8% to 22.3% in the women and 1.3% to 15.4% in the men [12]. Similarly, the data from the Dutch Lifelines cohort study, which included individuals aged 18 to 90 years, showed a global incidence of 1.4% in the women and 0.9% in the men, with the rates climbing to 16.7% among those aged 80 to 89 years [13]. Furthermore, a meta-analysis of 50 studies encompassing 86,285 participants reported a global incidence of sarcopenic obesity of 11% among adults aged 60 years and older [14]. The prevalence of both sarcopenia and sarcopenic obesity among the elderly highlights their link to increased all-cause mortality [15].

In today’s hyper-aged society, a thorough investigation into the causes of sarcopenia is essential for the successful development of preventive and therapeutic strategies.

Various approaches, including exercise, drug and nutritional supplementation, and hormone therapy, have been explored to mitigate muscle atrophy associated with sarcopenia [16]. Among these, nutritional therapy via supplementation with polyunsaturated fatty acids, high-quality protein, vitamins, and essential amino acids has been shown to prevent muscle weakness in elderly people by stimulating protein synthesis [17,18,19]. Soy foods are traditional Asian foods that alleviate muscle weakness by stimulating muscle protein synthesis and increasing antioxidant capacity [20]. Isoflavone, an abundant soybean flavonoid, has been studied in the context of preventing obesity, elevated blood sugar levels, osteoporosis, breast cancer, and its antioxidant properties in recent years [2]. However, the effectiveness of the soy isoflavones in preventing muscle atrophy in sarcopenia and sarcopenic obesity remains unclear.

Most soy isoflavones are glycosides, the sugars from which are removed by intestinal bacteria prior to absorption and become aglycons. Isoflavone aglycones, including daidzein and genistein, are further [21] metabolized by intracellular and gut bacterial enzymes to produce more active compounds [22,23].

Previous studies have shown that a high-fat, high-sucrose diet, akin to Western dietary patterns, induces chronic inflammation in visceral fat and results in metabolic disorders, including obesity, glucose intolerance, dyslipidemia, and sarcopenic obesity characterized by skeletal muscle atrophy [21,22]. Additionally, there are several reports on the association between the isoflavones and the prevention of muscle atrophy [24,25]. For instance, isoflavones exacerbated muscle atrophy in post-oophorectomy mice fed on a high-fat diet for 12 weeks [26]. Isoflavone treatment also markedly inhibited TNF-α-induced MuRF1 promoter activity and myotube atrophy in C2C12 myotubes [27]. Furthermore, daidzein has been reported to mitigate cisplatin-induced muscle atrophy [28] and increase skeletal muscle mass in young female mice [29].

Given that soy isoflavones have been shown to inhibit muscle atrophy, we hypothesized that these compounds may also prevent sarcopenia and obesity. Hence, the aim of this study was to investigate the effects of soy isoflavones on sarcopenic obesity and to examine the underlying mechanisms.

## 2. Materials and Methods

### 2.1. Murine Models

All procedures were approved by the Committee for Animal Research at the Kyoto Prefectural University of Medicine (Approval No. M2021-49). The study utilized male C57BL/6 J (WT) mice, aged seven weeks. Each cage housed six mice. The isoflavone group (Iso group) received water supplemented with 0.1% isoflavone ad libitum. Details regarding the procurement, housing, and feeding of the mice are provided in Data S1. The mice were euthanized at 20 weeks of age using a combination anesthetic comprising 5.0 mg/kg butorphanol, 4.0 mg/kg midazolam, and 0.3 mg/kg medetomidine, on account of ketamine being declared a narcotic in Japan in 2007.

### 2.2. Glucose and Insulin Tolerance Tests

Murine models, aged 20 weeks, underwent intraperitoneal glucose tolerance evaluations (iPGTT) (administered at 2 g/kg of body mass) after a fasting period of 16 h and were subjected to insulin tolerance assessments (ITT) (dispensed at 0.5 U/kg of body weight) following a 5 h fast. Details are provided in Data S1.

### 2.3. Assessment of Grip Strength

The grip strength was quantified using a specialized grip strength meter for murine models. Details are provided in Data S1.

### 2.4. Biochemistry

Cardiac puncture was performed under anesthesia in order to collect peripheral blood, and serum was separated by centrifugation. Alanine aminotransferase (ALT), total cholesterol, and triglyceride levels were subsequently measured. Details are provided in Data S1.

### 2.5. Histopathological Examination of Soleus and Plantaris Muscle Tissue

For the assessment of the soleus and plantaris muscles, the tissue samples were obtained from euthanized murine models for a histopathological assessment. Details are provided in Data S1.

### 2.6. Analysis of Gene Expression in the Soleus Muscle

RT-PCR was conducted to analyze gene expression in the soleus muscle. Details are described in Data S1.

### 2.7. Measurement of Daidzein, Genistein, and Equol Concentrations in Serum, Feces, and Soleus Muscle

Daidzein, genistein, and equol concentrations in murine serum, rectal feces, and soleus muscle samples were examined. Details are provided in Data S1.

### 2.8. Culture of Mouse Skeletal Muscle Cells

C2C12 cells (mouse myoblast cell line; KAC Co., Ltd., Kyoto, Japan) were cultured starting from day 0, with conditions detailed in Data S1. On day 7, cells were treated with either DMEM (Ctrl), 100 μM palmitic acid (PA), or 100 μM palmitic acid with 25 μM daidzein (PA + DZ) for 24 h after changing the differentiation medium. Myotubes were evaluated on day 8 under all experimental conditions.

### 2.9. Analysis of Gene Expression in C2C12 Myotube Cells

Gene expression in C2C12 myotube cells was evaluated on day 8 using RT-PCR. Details are described in Data S1.

### 2.10. Protein Extraction and Western Blot Analysis

Proteins were extracted from C2C12 cells and analyzed via Western blot. Details are provided in Data S1.

### 2.11. Statistical Analysis

Details of the statistical analysis are described in Data S1.

## 3. Results

### 3.1. The Administration of Isoflavone Reduced Body Weight and Ameliorated Glucose Intolerance Induced by the High-Fat High-Sucrose Diet (HFHSD)

**Figure 1:**
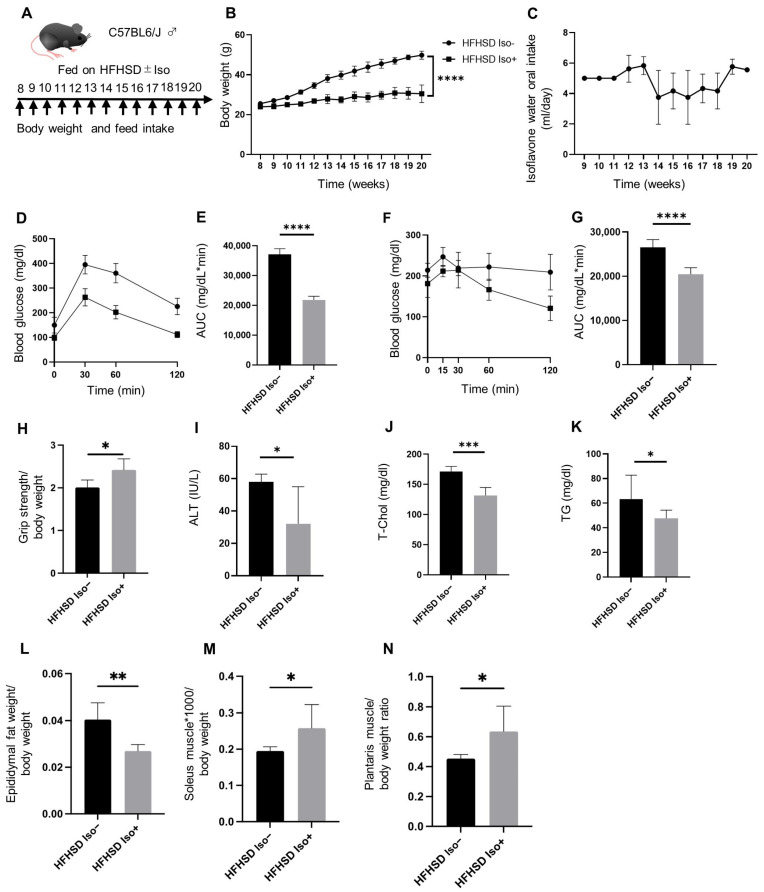
The *C57BL6/J* mice fed a high-fat high-sucrose diet (HFHSD) and administered water containing 0.1% isoflavone (Iso group) showed significant improvements in glucose tolerance, obesity, metabolic disorders, and muscle loss compared to the *C57BL6/J* mice without the isoflavone (Ctrl group). (**A**) Mice were fed HFHSD ± water containing 0.1% isoflavone for 12 weeks, starting at 8 weeks of age. (**B**) Changes in body weight (*n* = 6 in each case). (**C**) Oral intake of 0.1% isoflavone water. (**D**,**E**) The results of the intraperitoneal glucose tolerance test (2 g/kg body weight) for 20-week-old mice and the area-under-the-curve (AUC) analysis (*n* = 6 in each case). (**F**,**G**) The results of the insulin tolerance test (0.75 U/kg body weight) for the 20-week-old mice and the AUC analysis (*n* = 6 in each case). (**H**) Relative grip strength (*n* = 6 in each case). (**I**–**K**) Serum levels of alanine aminotransferase (ALT), total cholesterol (T-Chol), and triglycerides (TG) (*n* = 6 in each case). (**L**–**N**) The relative weight of the epididymal fat, soleus muscle, and plantaris muscle (*n* = 6 in each case). The data are expressed as mean ± standard deviation (SD). The data were analyzed using an unpaired *t*-test. * *p <* 0.05, ** *p <* 0.01, *** *p <* 0.001, **** *p* < 0.0001.

Significantly lower body weights were recorded in the mice fed an HFHSD along with isoflavones (Iso group) than those of mice fed an HFHSD alone (control group) (Figure 1B). Additionally, the area under the curve (AUC) for the blood glucose of the Iso group during the iPGTT and ITT was significantly lower than that of the Ctrl group (iPGTT, *p* < 0.0001; ITT, *p* < 0.0001) (Figure 1D–G). 

### 3.2. Administration of Isoflavone Reduced HFHSD-Induced Elevation of Liver Enzymes and Serum Lipid Levels

The Iso group exhibited significantly lower levels of serum alanine aminotransferase (ALT), triglycerides (TG), and total cholesterol than those exhibited by the control group (ALT, *p* = 0.0221; TG, *p* = 0.0941; total cholesterol, *p* < 0.0001) (Figure 1I–K).

### 3.3. Administration of Isoflavone Alleviated Sarcopenic Obesity

**Figure 2:**
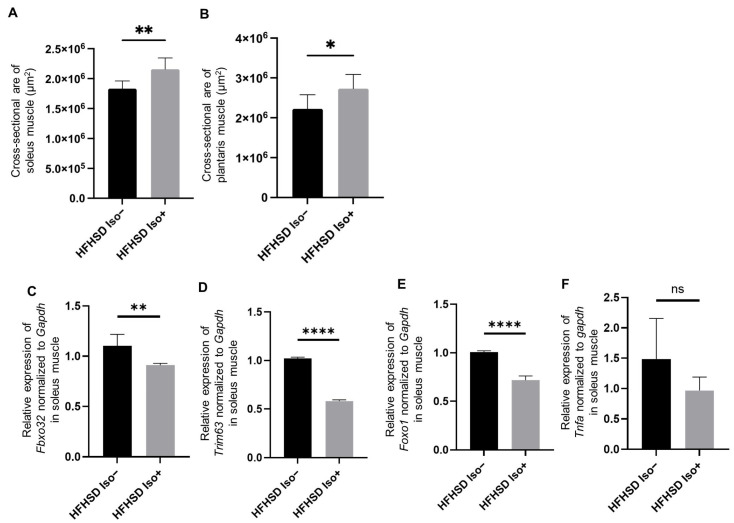
Effects of Isoflavone on muscle morphology and gene expression. (**A**,**B**) The cross-sectional areas of the soleus and plantaris muscles in the 20-week-old mice (*n* = 6 in each case). (**C**–**F**) The relative mRNA expressions of (**C**) *Fbxo32*, (**D**) *Trim63*, (**E**) *Foxo1*, and (**F**) *Tnfa* mRNA expression in the soleus muscle normalized to the expression of *Gapdh* (*n* = 6 in each case). The data are expressed as mean ± standard deviation (SD). The data were analyzed using an unpaired *t*-test. * *p <* 0.05, ** *p <* 0.01, **** *p* < 0.0001, ns not significant.

The Iso group showed a greater relative grip strength than that shown by the Ctrl group (*p* = 0.0460). Additionally, the cross-sectional areas of the soleus and plantaris muscles were larger in the Iso group than those in the Ctrl group (soleus muscle, *p* = 0.0060; plantaris muscle, *p* = 0.0320) (Figure 1H and Figure 2A,B). Furthermore, while the relative weight of epididymal fat in the Iso group was significantly reduced compared to that in the control group (*p* = 0.0020), the relative weights of the soleus and plantaris muscles in the Iso group were significantly higher than those in the Ctrl group (soleus muscle, *p* = 0.0410; plantaris muscles, *p* = 0.0260) (Figure 1L–N).

### 3.4. Administration of Isoflavone Decreased Expression of Muscle Atrophy and Inflammation-Related Genes in Skeletal Muscles

The relative expression of *Fbxo32*, *Trim63*, and *Foxo1* normalized to *Gapdh* in the soleus muscle was significantly lower in the Iso group than that in the control group. The relative expression of *Tnfa* in the soleus muscle tended to be lower in the Iso group than that in the control group (*Fbxo32*, *p* = 0.0012; *Trim63*, *p* < 0.0001; *Foxo1*, *p* < 0.0001; *Tnfa*, *p* = 0.1343) (Figure 2C–F).

### 3.5. Administration of Isoflavone Increased Serum, Fecal, and Muscle Daidzein Levels

**Figure 3:**
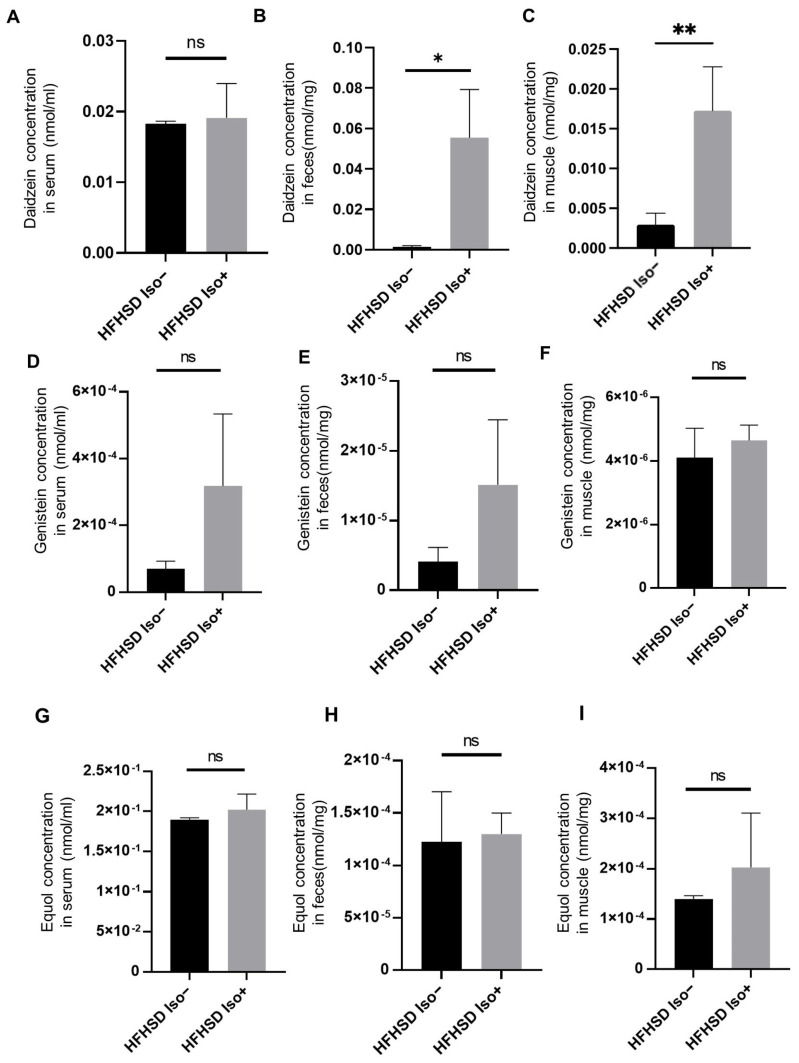
The administration of the isoflavone increased the concentration of the daidzein in the serum, feces, and muscles. The concentrations of the daidzein in (**A**) serum, (**B**) feces, and (**C**) soleus muscle (*n* = 6 in each group). The concentrations of the genistein in (**D**) serum, (**E**) feces, and (**F**) soleus muscle (*n* = 6 in each group). The concentrations of the equol in (**G**) serum, (**H**) feces, and (**I**) soleus muscle (*n* = 6 in each group). The data are expressed as mean ± standard deviation (SD). The data were analyzed using an unpaired *t*-test. * *p* < 0.05, ** *p* < 0.01, ns not significant.

The fecal and muscle daidzein levels were significantly higher in the Iso group than those in the control group (feces, *p* = 0.0122; muscle, *p* = 0.0020) (Figure 3B,C). Furthermore, the serum daidzein levels tended to increase in the Iso group compared to those in the control group (*p* = 0.8276) (Figure 3A). Genistein levels in serum, feces, and muscle were not significantly different between the Ctrl and Iso groups (serum, *p* = 0.0624; feces, *p* = 0.1159; muscle, *p* = 0.4198) (Figure 3D–F). Similarly, equol levels in serum, feces, and muscles did not differ significantly between the Ctrl and Iso groups (serum, *p* = 0.3324; feces, *p* = 0.8108; muscle, *p* = 0.2888) (Figure 3G–I).

### 3.6. Daidzein Decreased Palmitic Acid-Induced Expression of Inflammation-Related and Muscle Atrophy Genes in C2C12 Myotube Cells

**Figure 4:**
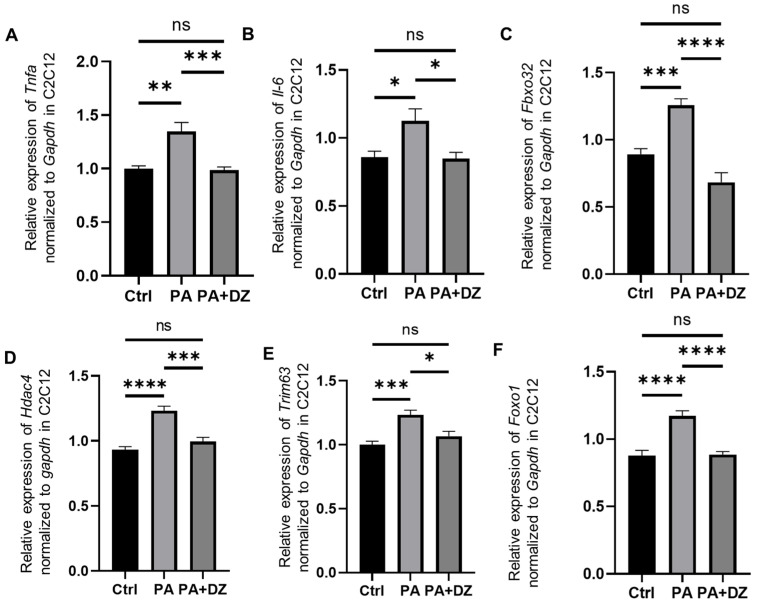
Impact of daidzein on gene expression in C2C12 myotube cells. The relative mRNA expression of (**A**) *Tnfa,* (**B**) *Il-6*, (**C**) *Fbxo32*, (**D**) *Hdac4*, (**E**) *Trim63*, and (**F**) *Foxo1* all normalized to the expression of *Gapdh* in the C2C12 cells (*n* = 6 in each case). The data were analyzed using a one-way ANOVA followed by Holm–Šídák’s multiple-comparisons test. * *p* < 0.05, ** *p* < 0.01, *** *p* < 0.001, **** *p* < 0.0001, ns not significant.

The expression of muscle atrophy- and inflammation-related genes in C2C12 cells, including *Tnfa*, *Il-6*, *Fbxo32*, *Hdac4*, *Trim63*, and *Foxo1,* was analyzed. The expression of these genes in the C2C12 cells supplemented with 100 μM PA was significantly higher than that of those in the C2C12 cells supplemented with DMEM (Ctrl) (*Tnfa*, *p* = 0.0253; *Il-6*, *p* = 0.0011; *Fbxo32*, *p* = 0.0009; *Hdac4, p* < 0.0001; *Trim63*, *p* = 0.0007; *Foxo1*, *p* < 0.0001). Conversely, the expression of these genes in the C2C12 cells supplemented with 100 μM PA and 25 μM daidzein (PA + DZ) was significantly lower than that of those in the PA group (*Tnfa*, *p* = 0.0201; *Il-6*, *p* = 0.0008; *Fbxo32*, *p* < 0.0001; *Hdac4*, *p* = 0.0002; *Trim63*, *p* = 0.0114; *Foxo1*, *p* < 0.0001) (Figure 4A–F).

### 3.7. Daidzein Reduced Palmitic Acid-Induced Expression of Muscle Atrophy Proteins in C2C12 Myotube Cells

**Figure 5:**
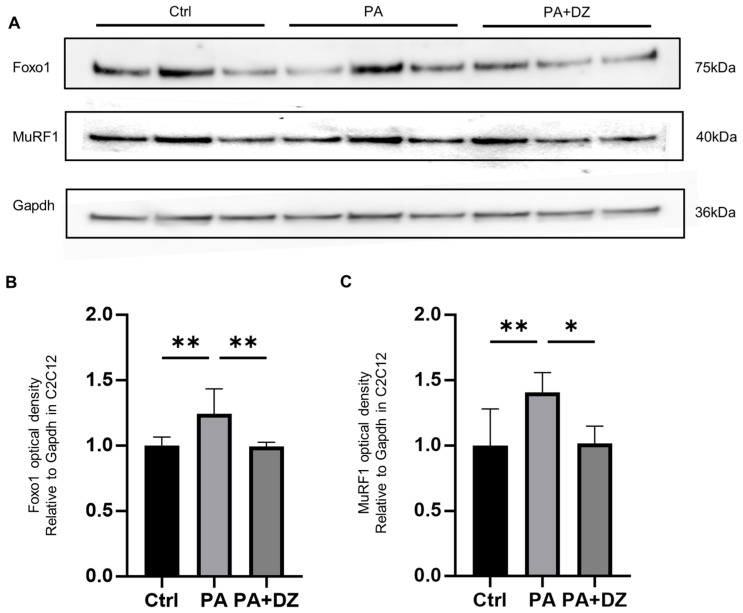
Effect of daidzein on muscle atrophy proteins in C2C12 myotube cells. (**A**) Western blot analysis depicting the levels of *Foxo1*, MuRF1, and *Gapdh* in the C2C12 myotube cells. (**B**,**C**) The relative optical density of *Foxo1* (**B**) and MuRF1 (**C**) normalized to *Gapdh*. The values are expressed as mean ± s.e.m. * *p*  <  0.05, ** *p*  <  0.01, as determined using a one-way ANOVA.

The expression levels of the muscle atrophy proteins in the C2C12 myotube cells, including *Foxo1* and MuRF1, were investigated. The expression levels of these proteins in the C2C12 cells supplemented with 100 μM PA were significantly higher than those of the proteins in the C2C12 cells supplemented with DMEM (Ctrl) (*Foxo1*, *p* = 0.0089; MuRF1, *p* = 0.0088). Conversely, the expression levels of the muscle atrophy proteins in the C2C12 cells supplemented with 100 μM PA and 25 μM daidzein (PA + DZ) were significantly lower than those of the proteins in the PA group (*Foxo1*, *p* = 0.0078; MuRF1, *p* = 0.0119) (Figure 5A–C).

## 4. Discussion

We demonstrated that soy isoflavones effectively prevented muscle atrophy induced by an HFHSD. Additionally, daidzein, a soy isoflavone, exhibited an inhibitory effect on muscle atrophy by suppressing the expression of the inflammatory cytokines and muscle atrophy genes in the myotubular cells. To the best of our knowledge, this study is the first to directly measure flavonoid levels, including daidzein, in skeletal muscle and to demonstrate their anti-muscle atrophy effects.

Previously, we reported that an HFHSD mimicking Westernized dietary patterns induces chronic visceral fat inflammation that results in metabolic disorders such as obesity, glucose intolerance, and dyslipidemia. Soy isoflavones have been shown to exert anti-inflammatory effects via innate lymphocytes in visceral fat, improving these metabolic conditions [23]. In sub-analyses, the mice fed with a high-fat, high-sucrose diet developed sarcopenic obesity accompanied by skeletal muscle inflammation, muscle atrophy, and visceral fat obesity. In this study, the isoflavone administration halted the HFHSD-induced weight gain and glucose intolerance, reduced the HFHSD-induced elevation of liver enzymes and serum lipid levels, and alleviated various metabolic disorders. These findings are consistent with previous reports that soy proteins and isoflavones effectively lower liver and blood lipids, improve glucose tolerance and insulin sensitivity, and reduce hepatic lipidosis [30].

An HFHSD has been shown to increase inflammation, as evidenced by elevated *Tnfα* expression in muscle [31], leading to muscle atrophy [32]. In contrast, soy isoflavones are known for their potent anti-inflammatory activities [33] and ability to inhibit muscle atrophy [34].

In this study, the isoflavone administration enhanced relative grip strength and increased the weights of the soleus and plantaris muscles in mice. It also decreased the expression of the muscle atrophy- and inflammation-related genes in skeletal muscle. These results suggest that soy isoflavones ameliorate obesity, metabolic function, and muscle atrophy induced by an HFHSD. Among soy isoflavones, daidzein has garnered considerable interest in recent years due to its broad therapeutic effects on oxidative stress, inflammation, obesity, neuroprotection, diabetes, anxiety, cardiovascular disease, cancer, and ovariectomy [35]. We observed that the isoflavone administration significantly increased the fecal and muscular daidzein levels, with a less dramatic effect on the serum daidzein levels. Collectively, these findings support the hypothesis that the daidzein may contribute to the inhibitory effects exerted by soy isoflavones.

Most soybean isoflavones are glycosides, which are not readily absorbed by the body. Efficient absorption mandates the removal of sugar from the glycoside to produce aglyconylated isoflavones [36]. This process is facilitated by cellular β-glucosidases derived from gut flora [37]. Isoflavone aglycones, including daidzein and genistein, are absorbed via the intestinal tract and are further metabolized by cellular and gut flora-derived enzymes to produce more biologically active compounds such as equol [38].

Among the flavonoids, we focused on daidzein on account of its significant elevation in muscle and fecal concentrations. Apart from its role in ameliorating cisplatin-induced muscle atrophy, the compound has been reported to decrease the expression of the ubiquitin-specific protease 19 via estrogen receptor beta and increase skeletal muscle mass in young female mice [28].

We therefore performed cellular experiments using mouse C2C12 myotubes to elucidate the underlying mechanisms of daidzein-mediated inhibition of muscle atrophy. PA, a major circulating long-chain saturated fatty acid, is often used as an in vitro high-fat inducer, prompting daidzein to reverse a palmitate-induced expression of inflammatory cytokines (*Tnfa* and *Il-6*) and muscle atrophy genes (*Fbxo32*, *Hdac4*, *Trim63*, and *Foxo1*) in the mouse C2C12 myotubular cells. This reversal was further confirmed for *Foxo1* and MuRF1 protein expression by Western blotting.

Daidzein has been shown to protect against palmitate-induced oxidative stress and apoptosis via PGC1α [39]. It also mitigates cisplatin-induced muscle atrophy by modulating the Glut4/AMPK/FoxO signaling pathway [28] and inhibits muscular lipid deposition while reducing the lipotoxicity in muscle by regulating the estrogen-related receptor alpha (ERRα) pathway [40]. Additionally, the soy isoflavones significantly suppress muscle atrophy by inhibiting the TNF-α-induced MuRF1 transcriptional activity in the C2C12 myotube cells [41]. We observed that daidzein inhibited muscle atrophy by suppressing the inflammatory cytokines and muscle atrophy genes upregulated by PA, both in vitro and in vivo. This effect may be mediated via multiple pathways, including the ubiquitin proteasome-dependent proteolytic pathway, PGC1α, Glut4/AMPK/FoxO, and the ERRα pathway.

In the present study, isoflavone was converted into its aglycon form, daidzein, in the intestine, resulting in increased daidzein concentrations in skeletal muscle. This likely contributes to the prevention of glucose intolerance and skeletal muscle atrophy by suppressing the expression of the inflammatory cytokine and muscle atrophy genes.

For some people, sarcopenia and sarcopenic obesity can be reversed or their progression can be slowed or halted through an appropriate diet and exercise therapy [42]. Human studies have shown that soy isoflavones reduce TNF-α levels and induce anti-inflammatory effects, potentially offering pronounced benefits for patients with sarcopenia [43]. The mice in this study ingested 167 ng/g/day of isoflavones at 20 weeks of age, equivalent to 5 μg/day. This is comparable to an intake of 10 mg/day for a person weighing 60 kg. Isoflavones are found in soy products, including natto, tofu, and miso soup, that are often consumed in Japan. Given the isoflavone content of these foods [44,45,46], a 60 kg person should consume 1/4 pack of natto, 1/10 pack of tofu, and 1 1/2 cups of miso soup. The consumption of these soy products is expected to suppress sarcopenia and sarcopenic obesity.

This study is pioneering in directly measuring the flavonoid concentrations in skeletal muscle and demonstrating their anti-muscle atrophy effects. However, it has several limitations. While the in vivo administration of isoflavones was performed, specific experiments involving daidzein administration were not. Additionally, although the variations in the composition of the gut microbiota, which are crucial for the metabolism and action of isoflavones, are known to exist, detailed evaluations of the gut microbiota among individual mice were not carried out. Furthermore, this study did not provide sufficient details to relate the test concentrations of soy isoflavones used to levels that demonstrate usefulness in humans. Additional research is needed to determine whether the isoflavone concentrations used in in vitro studies can be physiologically achieved under in vivo conditions.

## 5. Conclusions

Daidzein, an isoflavone, effectively inhibited muscle atrophy by suppressing the expression of inflammatory cytokine- and muscle atrophy-related proteins. This study sheds new light on the muscle atrophy suppression capabilities of soy isoflavones, particularly daidzein, highlighting their potential in preventing and managing sarcopenia and sarcopenic obesity. Therefore, our findings are considered to be of significant importance for the prevention of sarcopenia and sarcopenic obesity through the dietary intake of soy foods.

## Data Availability

The original contributions presented in the study are included in the article/Supplementary Material; further inquiries can be directed to the corresponding author/s.

## Supplementary Materials

The following supporting information can be downloaded at: https://www.mdpi.com/article/10.3390/nu16183084/s1, Data S1: The additional details concerning Materials and Methods.
