# Supplementary material for: Daidzein Inhibits Muscle Atrophy by Suppressing Inflammatory Cytokine- and Muscle Atrophy-Related Gene Expression
Source: Nutrients. 2024 Sep 13;16(18):3084. doi: 10.3390/nu16183084 (PMC11434955; doi:10.3390/nu16183084)
Supplement: Supplementary file 1 [file nutrients-16-03084-s001.zip › nutrients-3137382-supplementary.pdf]

Methods

Murine Models

The Committee for Animal Research of the Kyoto Prefectural University of Medi-cine approved all experimental procedures (Approval No. M2021-49). Seven-week-old C57BL/6 J (WT) male mice were procured from Shimizu Laboratory Supplies (Kyoto, Japan), and housed in a specific pathogen-free controlled environment at 23 ± 1.5 °C with a 12-h light/12-h dark cycle (7 a.m.-7 p.m.). Mice were housed in cages of W220 × L320 × H135, with six mice in each cage. Experimental procedures involved admin-istration of a high-fat, high-sucrose diet (HFHSD; 459 kcal/100 g, 20% protein, 40% carbohydrate, and 40% fat; D12327, Research Diets, Inc., New Brunswick, NJ, USA) for 12 weeks, which was initiated at 8 weeks of age. The isoflavone group (Iso group) was administered water supplemented with 0.1% isoflavone ad libitum. Equal amounts of feed were supplied to ensure pair feeding, and body weight and oral intake were measured weekly. Mice were sacrificed at 20 weeks of age by administering a combi-nation anesthetic comprising 0.3 mg/kg medetomidine, 4.0 mg/kg midazolam, and 5.0 mg/kg butorphanol on account of ketamine being declared a narcotic in Japan in 2007.

Glucose and insulin tolerance tests

Intraperitoneal glucose tolerance tests (IPGTT; 2 g/kg body weight) and insulin tolerance tests (ITT; 0.5 U/kg body weight) were performed on 20-week-old mice fol-lowing fasting periods of 16 hours and 5 hours, respectively. Blood glucose concentra-tions were measured at 0, 30, 60, and 120 minutes post-injection for the IPGTT, and at 0, 15, 30, 60, and 120 minutes for the ITT using a glucometer (Gultest Neo Alpha; Sanwa Kagaku Kenkyusho, Nagoya, Japan). The areas under the curve (AUC) for both the IPGTT and ITT were subsequently calculated.

Assessment of grip strength

Grip strength was evaluated using a strength meter (model DS2-50N; IMADA Co., Ltd, Toyoashi, Japan) in 20-week-old mice. Six consecutive measurements were rec-orded daily at 1-minute intervals, and grip strength was standardized according to body weight. All investigators were blinded to the experimental conditions.

Biochemistry

Cardiac puncture was performed under anesthesia in order to collect peripheral blood, and serum was separated by centrifugation at 4,000 rpm for 10 minutes at 4 °C. Alanine aminotransferase (ALT), total cholesterol, and triglyceride levels were subse-quently

measured using reagents procured from FUJIFILM Wako Pure 18 Chemical Corporation (Osaka, Japan).

Histopathological Examination of Soleus and Plantaris Muscle Tissue

The soleus and plantaris muscles were fixed in 10% buffered formaldehyde and embedded in paraffin. Muscle sections were stained with hematoxylin and eosin. Im-ages were acquired using a BZ-X710 fluorescence microscope (Keyence, Osaka, Japan), and cross-sectional areas were quantified using ImageJ software (NIH).

Analysis of gene expression in the soleus muscle

RT-PCR was performed to assess gene expression in soleus muscle. Soleus muscle was homogenized in ice-cold QIAzol Lysis reagent (Qiagen, Hilden, Germany), and total RNA was isolated according to the manufacturer's instructions and measured using NanoDrop (Thermo Fisher Scientific). Total RNA (0.5 µg) was reverse-transcribed using a High-Capacity cDNA Reverse Transcription Kit (Applied Biosystems, Foster City, CA, USA) for first-strand cDNA synthesis by using an oligonucleotide dT primer and random hexamer priming, according to the manufacturer's recommendations. Reverse transcription reactions were performed at 37°C for 120 min and reverse transcription at 85°C for 5 min. Real-time reverse transcription-polymerase chain reaction (RT-PCR) was used to quantitate the mRNA expression levels of Tnfa, Fbxo32, Trim63, and Foxo1 in the muscle. RT-PCR was performed using TaqMan Fast Advanced Master Mix (Applied Biosystems), according to the manufacturer's instructions. The following PCR conditions were used: 1 cycle of 2 min at 50°C and 20 s at 95°C, followed by 40 cycles of 1 s at 95°C, and 20 s at 60°C. The relative expression levels of each targeted gene were normalized to the Gapdh threshold cycle (CT) values and quantified using the comparative threshold cycle $2^{-\Delta\Delta CT}$ method, as previously described. Signals from Ctrl group were assigned a relative value of 1.0. Samples from 6 mice from each group were examined, and RT-PCR was performed in triplicate for each sample.

Measurement of daidzein, genistein and equol concentrations in serum, feces, and soleus muscle

The daidzein concentrations of the murine serum, rectal feces, and soleus muscle samples was analyzed using gas chromatography (GC) mass spectrometry (MS) on an Agilent 7890B/7000D System (Agilent Technologies, Santa Clara, CA, USA). Serum (50 µL), rectal feces (20 mg), and soleus muscle (20 mg) samples were added to 500 µL acetonitrile and 500 µL diluted water and ground in a ball mill at 4000×rpm for 2 min.

Next, the samples were shaken at 1000× rpm for 30 min at 37°C and centrifuged at 14,000×rpm for 3 min at room temperature. The supernatant (500 μL) was separated, added to 500 μL acetonitrile, and further shaken at 1000× rpm for 3 min at 37°C. The samples were then centrifuged at room temperature for 3 min at 14,000×rpm, and the pH was adjusted to 9 with 0.1 mol/L NaOH to extract the flavonoids. The daidzein concentration was then automatically determined by GC/MS using the online solid-phase extraction (SPE) method. In the SPE-GC system SGI-M100 (AiSTI Science, Wakayama, Japan), SPE and injection into the GC/MS system are automatically performed after the sample is added to the vial and set on the autosampler tray. Flash SPE ACXs (AiSTI Science) were used for solid stratification. Fifty-microliter aliquots of each of the aforementioned sample extracts were obtained, loaded onto the solid phase, and washed with acetonitrile and water (1:1). Next, the samples were dehydrated with acetone, impregnated with 4 μL N-tert-butyldimethylsilyl-N-methyltrifluoroacetamide (MTBSTFA)-toluene solution (1:3), and eluted with hexane after derivatization on the solid phase. The final product was injected using a programmed temperature vaporizer (PTV) injector, LVI-S250 (AiSTI SCIENCE), whose temperature was maintained at 150°C for 0.5 min, increased gradually from 25°C/min to 290°C, and then maintained there for 16 min. The samples were loaded onto a capillary column, Vf-5ms (30 m × 0.25 mm (inner diameter0 × 0.25 μm (membrane thickness); Agilent Technologies). The column temperature was maintained at 60°C for 3 min, increased gradually by 10 °C/min to 100°C and subsequently by 20°C/min to 310°C, and then maintained at 310°C for 7 min.

Culture of Mouse Skeletal Muscle Cells

C2C12 cells (mouse myoblast cell line; KAC Co. Ltd., Kyoto, Japan) were seeded at a density of 1 x 104 cells per well in 96-well plates and cultured in Dulbecco's modified Eagle's medium (DMEM) supplemented with 20% fetal bovine serum at 37°C in a humidified atmosphere with 5% CO2 (day -4). The culture medium included 1% penicillin-streptomycin. Medium change was performed every other day. Upon reaching 80% confluence, cells were differentiated in DMEM supplemented with 2% horse serum (differentiation medium) (day 0). On day 7, cells were treated with either DMEM without any additional compounds (Ctrl), 100μM palmitic acid (PA), or 100μM palmitic acid with 25μM daidzein(PA+DZ) for 24h, post changing differentiation medium. Myotube were evaluated for all experimental conditions on day 8.

Analysis of gene expression in C2C12 Myotube Cells

Gene expression in C2C12 cells was evaluated on day 8. Following the removal of the culture medium, the cells were detached using pipettes and subsequently homoge-nized in ice-cold QIAzol Lysis Reagent. Total RNA was then extracted according to the manufacturer's protocol. The isolated RNA was reverse-transcribed into complemen-tary DNA (cDNA) using a High-Capacity cDNA Reverse Transcription Kit (Applied Biosystems), with oligo-dT and random hexamer primers, following the manufactur-er's instructions. The reverse transcription reaction was conducted for 120 minutes at 37 °C, and the reaction was terminated by heating at 85 °C for 5 minutes. The mRNA levels of Tnfa, Il6, Fbxo32, Hdac4, Trim63, and Foxo1, which are associated with muscle atrophy, were quantified via real-time reverse transcription polymerase chain reaction (RT-PCR). RT-PCR was carried out using the TaqMan Fast Advanced Master Mix (Ap-plied Biosystems) under the following thermal cycling conditions: one cycle of 2 minutes at 50 °C and 20 seconds at 95 °C, followed by 40 cycles of 1 second at 95 °C and 20 seconds at 60 °C. The relative expression of each target gene was normalized to the threshold cycle (CT) value of Gapdh using the comparative 2−ΔΔCT method as previously described. Each sample was analyzed in triplicate.

Protein extraction and Western blot analysis

Whole C2C12 myotube cell lysates were prepared using a radioimmunoprecipita-tion assay (RIPA) buffer (ATTO, Tokyo, Japan; 50 mmol/L Tris (pH 8.0), 150 mmol/L NaCl, 0.5% deoxycholate, 0.1% SDS, and 1.0% NP-40) supplemented with a protease inhibitor cocktail (BioVision, Milpitas, CA, USA). Protein concentrations were deter-mined using a bovine serum albumin (BSA) protein assay kit (Pierce, Thermo Scien-tific) following the manufacturer's instructions. Total protein (40 μg) was separated on 12% SDS-PAGE gels, and Western blotting was performed according to standard pro-tocols. Protein detection was carried out using the ImageQuant LAS 500 system (GE Healthcare). Quantification of protein expression levels was achieved by measuring optical density with ImageJ software, with the optical densities of Foxo1 and MuRF1 normalized to that of Gapdh to facilitate comparative analysis of their expression lev-els across samples.

Protein extracts containing 40–60 μg of protein were incubated overnight at 4°C with the following primary antibodies: Foxo1 (1:1000, Proteintech, Rosemont, IL, USA), MuRF1 (1:1000, Proteintech), or Gapdh (1:1000, Proteintech), diluted in EzBlock Chemi (ATTO, Osaka, Japan). Subsequently, the membranes were incubated with goat an-ti-rabbit IgG secondary antibodies conjugated to horseradish peroxidase (Bio-Rad, Hercules, CA, USA), diluted in EzBlock Chemi for 30 minutes at room temperature. All antibodies used in this study were obtained from Santa Cruz Biotechnology (Santa Cruz, CA, USA).

Statistical analysis

Data analysis was performed using GraphPad Prism (version 14.0; San Diego, CA, USA). Welch's t-test was applied for comparisons between two groups, while one-way ANOVA followed by Holm–Šídák's multiple comparisons test was used for analysis across three groups. Statistical significance was defined as $p < 0.05$. All figures were generated using GraphPad Prism.
